# Preload time-dependent effects of *Panax ginseng* on postprandial glucose tolerance. A randomized controlled study in healthy middle-aged participants

**DOI:** 10.3389/fnut.2026.1759162

**Published:** 2026-02-20

**Authors:** Anne Nilsson

**Affiliations:** Department of Process and Life Science Engineering, Division Food and Pharma, Lund University, Lund, Sweden

**Keywords:** *Panax ginseng*, preload timing, postprandial glucose regulation, metabolic health, postprandial insulinaemia, randomized controlled crossover trial

## Abstract

**Objectives:**

*Panax ginseng* (*P. ginseng*, C.A. Meyer) is suggested to improve blood glucose regulation, but evidence remains inconsistent. We examined whether consuming *P. ginseng* as a preload before a carbohydrate-rich meal modulates postprandial glucose and insulin responses.

**Methods:**

In a randomized, double-blind, crossover trial, 22 healthy middle-aged adults consumed *P. ginseng* tablets containing 32 mg total ginsenosides (primarily Rg1, Re, Rf, Rb1, Rc, Rb2, and Rd) or an identical placebo without ginsenosides at three time points prior to a standardized breakfast: 90 min, 45 min, and immediately before meal initiation.

**Results:**

Compared with placebo, *P. ginseng* ingested 90 min prior to the meal reduced 0–150 min postprandial blood glucose incremental areas under the curves (iAUC) by 29% (*P* < *0.001*) and maximum individual glucose peaks (iPeak) by 26% (*P* < *0.001*). When *P. ginseng* was ingested 45 min prior to the meal, postprandial iPeaks were reduced by 15% (*P* < *0.01*). Postprandial insulin iAUCs decreased by 24% (*P* < *0.001*) and 23% (*P* < *0.01*) when ginseng was consumed 90 and 45 min, respectively, before the meal. Similarly, compared with placebo, the insulin iPeaks were significantly lower after ginseng intake at 90 or 45 min prior to the meal (−23%, *P* < 0.001 and −15%, *P* < 0.01, respectively). No effects on blood glucose or insulin responses were observed when ginseng was taken immediately before the meal (*P* > *0.05*).

**Conclusion:**

Preload timing is critical for optimizing ginseng's glucoregulatory effects, supporting its potential role in dietary strategies for glycemic management.

Trial registered at ClinicalTrials.gov (NCT02392819). URL:clinicaltrials.gov/study/NCT02392819.

## Introduction

Diet is the most significant lifestyle factors in the development of obesity, type 2-diabetes (T2D) and cardiovascular diseases (CVD). Certain foods, such as dietary fiber (DF) (1) and whole grains (1), as well as legumes (2) have demonstrated preventive potential in this respect. Additionally, the concept of low glycaemic index (GI) and glycaemic load is highly relevant, as exaggerated postprandial glucose excursions are implicated in the pathogenesis of T2D and CVD (3–5). Consequently, dietary strategies aimed at attenuating postprandial blood glucose increments are considered important for the primary prevention of cardiometabolic diseases (CMD).

Several food characteristics contribute to a low GI, including intact botanical structures (e.g., kernel-based foods), the presence of viscous DF, and processing methods that limit starch accessibility to amylase. Beyond structural properties, specific food concepts have been proposed to reduce postprandial glycaemia, such as meal preloads rich in insulinogenic amino acids or proteins (6), and foods containing bioactive compounds like polyphenols and spices, for example cinnamon (7) and turmeric (8).

Ginseng (genus *Panax*), a medicinal herb traditionally associated with health benefits, has been used for thousands of years in East Asia. Both Asian (Korean) ginseng [*Panax ginseng* (*P. ginseng*)] and American ginseng [*Panax quinquefolius* (*P. quinquefolius*)] have been suggested to exert anti-diabetic effects, as reflected by improvements in fasting (9) and postprandial blood glucose concentration (10), as well as anti-inflammatory properties (11). However, findings are inconsistent. For instance, a study involving 60 participants with metabolic syndrome reported no significant effects on metabolic risk markers after daily intake of 4.5 g *P. ginseng* for 12 weeks (12). In contrast, a 2-week double-blind, placebo-controlled trial with fermented red ginseng (2.1 g/day) in 93 post-menopausal women with hypertension and/or T2D showed improved markers of glucose regulation, including reductions in glycosylated hemoglobin (HbA1c), insulin concentrations, and homeostatic model assessment of insulin resistance (HOMA-IR) (13). Similarly, a 12-week placebo-controlled intervention in 49 middle aged pre-diabetic patients revealed reduced HbA1c and postprandial glucose tolerance after daily intake of Red Ginseng Extract Powder (500 mg), with greater effects after 12 weeks compared with 6 weeks (14).

Regarding postprandial glucose excursions, previous evidence suggests that the timing of ginseng or ginsenoside intake may critically influence its efficacy. In a randomized crossover study of 12 healthy individuals, *P. quinquefolius* (1, 2, and 3 g) significantly reduced postprandial glucose concentrations following a 25 g glucose challenge when administered 40 min prior to the load, but not when ingested immediately before (15). Nevertheless, randomized controlled trials examining postprandial effects of ginseng remain limited, and studies addressing preload timing are particularly scarce.

Therefore, the objective of the present study was to investigate the time-dependent effects of a *P. ginseng* preload on markers of postprandial glucose regulation, specifically blood glucose and serum insulin concentrations. To this end, we conducted a double-blind, randomized, placebo-controlled crossover trial in 22 healthy middle-aged participants. The intervention assessed the effects of *P. ginseng* (32 mg total ginsenosides) administered at three different preload time points: (1) 90 min before breakfast, (2) 45 min before breakfast, and (3) immediately prior to breakfast.

## Materials and methods

### Test participants

Twenty-two healthy participants, 16 women and six men, with normal BMI (mean ± SD: 23.6 ± 1.8 kg/m^2^) and aged 52.8 ± 5.8 (mean ± SD) years were enrolled in the study (Table 1). All participants completed the study. The inclusion criteria were age between 40–60 years, BMI between 19–25 kg/m^2^, non-smoker and no known metabolic or cardiovascular diseases or food allergies. Health status was assessed based on self-reported health status and medical history obtained during screening. Participants reporting diagnosed hypertension, type 2 diabetes, dyslipidemia, or use of medication known to affect glucose metabolism were not eligible for inclusion. In addition, all participants displayed fasting glucose, insulin, and HOMA-IR values within the normal range (Table 1), supporting that the cohort was metabolically healthy.

**Table 1:** Characteristics of the participants^a^.

**Characteristics**	**Total cohort**	**Male**	**Female**
*n*	22	6	16
Age, years	52.8 ± 5.8	53.3 ± 4.8	52.6 ± 6.2
BMI, kg/m^2^	23.6 ± 1.8	24.6 ± 1.9	22.6 ± 1.4
Fasting glucose (mmol/L)	5.3 ± 0.4	5.3 ± 0.4	5.4 ± 0.4
Fasting insulin (nmol/L)	0.038 ± 0.019	0.034 ± 0.019	0.039 ± 0.019
HOMA-IR^b^	1.52 ± 0.87	1.35 ± 0.80	1.58 ± 0.89

^a^All values are mean ± SD.

^b^HOMA-IR: homeostatic model of insulin resistance coloring.

In addition to following a diet consistent with the Nordic Nutrition Recommendations (non-vegetarian), participants were instructed to maintain their habitual diet throughout the trial and to abstain from initiating any new dietary regimens. The use of dietary supplements, including multivitamins/minerals, herbal preparations, and specifically antioxidant-containing supplements (e.g., vitamin C, vitamin E, carotenoids, polyphenol concentrates), was not permitted within 2 weeks prior to the first test day nor during the study period. Participants confirmed compliance via self-report at screening and prior to each test day.

No antibiotics or probiotics were allowed within 2 weeks before or during the entire study period.

Prior to inclusion, each subject received a full written and oral explanation of the purpose and procedure of the study, and written informed consent was obtained from each participant.

This trial is reported in accordance with the CONSORT 2010 guidelines, including the extension for randomized crossover trials (Supplementary File 1). The completed CONSORT checklist is provided as Supplementary File 2.

### The test and placebo products

The ginseng test product consisted of a commercially available tablet containing *Panax ginseng* extract (C.A. Meyer), supplied by Orkla Health (ORKLA ASA, Oslo, Norway). Each tablet (200 mg) corresponded to 700 mg of ginseng root. The *Panax ginseng* extract was standardized for total ginsenoside content, with the pre-dominant ginsenosides characterized. The extraction solvent (aqueous or alcohol-based) was not specified by the manufacturer.

The total ginsenoside content and the pre-dominant ginsenosides (Rg1, Re, Rf, Rb1, Rc, Rb2, and Rd) were quantified by an independent contract laboratory using high-performance liquid chromatography (HPLC; Table 2). Analyses were performed on the study lot, and the mean ginsenoside content was 16 mg per tablet, corresponding to a total dose of 32 mg ginsenosides (two tablets) on test days with ginseng. The study dose corresponds to the labeled daily intake for this product category and was selected to evaluate effects at a translational, real-world exposure. The tablets were convex, film—coated, reddish-brown, and 13.5 mm in diameter. The placebo consisted of tablets identical in size, shape, and color to the ginseng tablets (13.5 mm, convex, reddish-brown, film-coated) but without ginseng extract. Thus, neither participants nor research personnel could distinguish between ginseng and placebo tablets. Apart from the ginseng extract in the *P. ginseng* tablets, both tablet types contained identical excipients: dicalcium phosphate (bulking agent), maltodextrin, maize starch, glazing agents (magnesium salts of fatty acids, hydroxypropyl methylcellulose, carnauba wax), and iron oxides/hydroxides for coloring.

**Table 2:** Contents of the most abundant ginsenosides in one ginseng tablet (200 mg)^a^.

**Sample**	**Rg1**	**Re**	**Rf**	**Rb1**	**Rc**	**Rb2**	**Rd**	**Total**
Sample 1 (mg)	2.00	5.49	0.18	1.12	3.27	1.39	2.69	16.14
Sample 2 (mg)	1.99	5.44	0.15	1.12	3.24	1.36	2.63	15.93

^a^The total ginsenoside content and the pre-dominant ginsenosides (Rg1, Re, Rf, Rb1, Rc, Rb2, and Rd) were quantified by an independent contract laboratory using high-performance liquid chromatography (HPLC).

Results are based on two replicates (sample 1 and 2).

### Study design and procedure

The study employed a randomized, double-blind, crossover design. Randomization was performed using a computer-generated sequence (Microsoft Excel). The trial was registered at ClinicalTrials.gov (Identifier: NCT02392819; registration date: March 2, 2015). Participant enrolment began on March 2, 2015, and the experimental phase was completed in July 2015. Each participant completed four experimental test days, separated by a minimum washout period of one week. A CONSORT flow diagram illustrating participant progression through enrollment, allocation, follow—up, and analysis is provided in Supplementary File 2.

On the evening prior to each test day, participants consumed a standardized evening meal at 21:00, consisting of white wheat bread and water, tea, or coffee (without milk or sugar). Following this meal, participants fasted overnight until arrival at the research facility at Lund University at 07:15 the next morning.

On each test day, participants ingested two tablets at three pre-defined preload time points aligned with the sampling schedule. The first pair was consumed at 0 min (fasting baseline), the second pair at 45 min, and the last pair at 90 min, immediately before the standardized breakfast was served. This means that breakfast corresponded to the 90-min time point in the sampling timeline. A total of six tablets were ingested per test day (two tablets per time point).

Across the four test days, one day served as an all—placebo control (placebo at 0, 45, and 90 min). On each of the remaining three days, *P. ginseng* tablets were administered at exactly one of these three time points (0, 45, or 90 min), while placebo tablets were given at the other two. This allocation ensured full blinding, as all tablets were identical in appearance. Participants consumed 100 ml of water with the tablets at each time point. The standardized breakfast was served at 09:00 and consisted of white wheat bread (providing 50 g available starch) and 200 ml water. Blood glucose was measured at 10 time points and serum insulin at eight time points over a 240 min period, extending to 150 min postprandially from the start of breakfast (see section below for exact timings).

**Figure 1:**
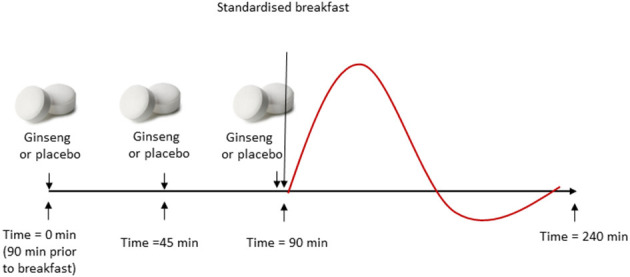
Study procedure during experimental days. Participants arrived in a fasting state at 07:30. Test variables were assessed at baseline (fasting) and repeatedly over 240 min. On each of four test days, participants ingested two tablets (ginseng or placebo) at three time points: 0 min (fasting), 45, and 90 min (immediately before breakfast). On three test days, ginseng was administered at one of these time points and placebo at the other two; on the remaining day, placebo was given at all three time points. A standardized breakfast was served at 90 min, immediately after tablet ingestion. Blood glucose and serum insulin were measured at 0 min (fasting), 45 min (glucose only), 90 min (start of breakfast), 105 min (glucose only), and at 120, 135, 150, 180, 210, and 240 min.

To minimize confounding from acute physical activity, participants were asked to avoid vigorous exercise and alcohol for 24 h prior to each test day and otherwise maintain their habitual activity level. On test mornings, participants remained seated at the research facility except for necessary activities. To increase within-subject standardization across the four crossover visits, participants recorded their lunch and dinner on the day before the first visit and replicated these meals prior to subsequent visits. Compliance with the pre-test restrictions (diet, supplements, physical activity, and alcohol) was confirmed by self-report upon arrival on each test day. Figure 1 illustrates the study procedure during the experimental days.

### Collection and analysis of glucose and insulin

Capillary blood samples were obtained via finger-prick for determination of blood glucose (HemoCue^®^ B-glucose, HemoCue AB, Ängelholm, Sweden) and serum insulin concentrations (Mercodia Insulin ELISA, Mercodia AB, Uppsala, Sweden). Measurements were performed at fasting (0 min), 45 min (glucose only), 90 min (prior to meal initiation), 105 min (glucose only), and subsequently at 120, 135, 150, 180, 210, and 240 min. Homeostatic Model Assessment of Insulin Resistance (HOMA-IR) (16) was calculated at baseline using fasting plasma glucose and serum insulin values (see Table 1).

### Calculation and statistical methods

The effects of *P. ginseng* on postprandial glucose tolerance and insulin concentrations were evaluated using incremental area under the curve (iAUC) and maximum incremental peak concentrations (iPeak), based on changes from baseline (fasting) values. iAUC was calculated for two time intervals: the entire experimental period (0–240 min) and the postprandial period following the standardized breakfast (90–240 min) for each subject and each test occasion, using the trapezoidal rule. iPeak was defined as the maximum postprandial increase from baseline for each subject and test occasion.

Model assumptions were evaluated prior to analysis. Normality of residuals was assessed using normal probability plots and histograms; residuals vs. fitted values and residuals vs. observation order were examined to assess homoscedasticity and independence. When indicated by visual inspection, the Anderson–Darling test was applied. Model assumptions were met.

Graphical representation of incremental curves and calculation of areas were performed in GraphPad Prism (version 6; GraphPad Software, San Diego, CA, USA). To provide a comprehensive overview, analyses based on absolute (actual) blood glucose and serum insulin concentrations were also conducted; these results are presented in Supplementary File 3.

Treatment effects on test variables were assessed using analysis of variance (ANOVA; general linear model) followed by Tukey's *post hoc* test for pairwise comparisons in MINITAB (release 17; Minitab Inc., State College, PA, USA). To examine treatment^*^ time interactions, repeated-measures mixed models were applied (PROC MIXED, SAS release 9.3; SAS Institute Inc., Cary, NC, USA) with an autoregressive covariance structure. As the study was not powered to detect sex-specific effects and the number of male participants was small (*n* = 6), sex was not included as a factor in the statistical models. The crossover design allowed participants to serve as their own controls, thereby minimizing the influence of between-subject factors on treatment effects.

The primary outcome was the change in blood glucose concentrations (iAUC) after the standardized breakfast, reflecting effects on glucose tolerance. Sample size was estimated in MINITAB based on data from a previous study investigating Korean red ginseng root fractions on glucose responses (iAUC 0–120 min) (17). Assuming a mean difference of 33 mmol^*^min/L between ginseng and control (cornstarch), a standard deviation of 45 mmol^*^min/L, α = 0.05, and power (1–β) = 0.8, 17 participants were required. To account for potential dropouts, the sample size was increased to 22.

Baseline characteristics are presented as means ± SD, while all other results are expressed as means ± SEM. Statistical significance was set at *P* < 0.05.

## Results

### Blood glucose

**Figure 2:**
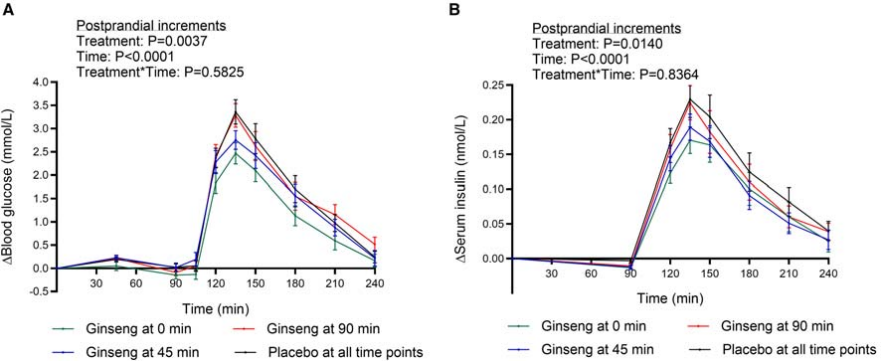
**(A)** Incremental blood glucose- and **(B)** serum insulin concentrations 0–240 min when placebo was consumed at all preload timepoints (placebo at fasting (0 min), 45 and 90 min), when *Panax ginseng* were provided at 0 min (placebo at 45 and 90 min), at 45 min (placebo at 0 and 90 min), and at 90 min (placebo at 0 and 45 min). A standardized breakfast was consumed directly after the intake of the tablets at 90 min. Concentrations are presented as mean ± SEM, *n*=22.

Significant main effects of *P. ginseng* treatment on incremental blood glucose concentrations were observed during the postprandial period (90–240 min; *P* < 0.01), with no significant treatment^*^time interaction (*P* > 0.05; Figure 2A, Table 3).

**Table 3:** Fasting and incremental blood glucose responses under four experimental conditions: (1) placebo consumed at all preload time points (0, 45, and 90 min); (2) *Panax ginseng* administered at 0 min (with placebo at 45 and 90 min); (3) *Panax ginseng* administered at 45 min (with placebo at 0 and 90 min); (4) *Panax ginseng* administered at 90 min (with placebo at 0 and 45 min)^1^.

**Variable**	**Placebo at all test points**	**Ginseng at 0 min**	**Ginseng at 45 min**	**Ginseng at 90 min**
Fasting glucose concentrations (mmol/L)^2^	5.3 ± 0.08^a^	5.5 ± 0.09^a^	5.3 ±0.09^a^	5.3 ±0.08^a^
90 min (mmol/L)^3^	0.02 ± 0.09^a^	−0.15 ± 0.08^a^	0.02 ± 0.08^a^	−0.09 ± 0.08^a^
iAUC 0–240 min (mmol^*^min/L)^4^	253.7 ± 25.6^a^	178.6 ± 20.8^b^	233.1 ± 21.6^a^	246.4 ± 28.3^a^
iAUC 90–240 min (mmol^*^min/L)^5^	236.9 ± 24.6^a^	169.0 ± 19.0^b^	208.5 ± 20.4^ab^	240.5 ± 26.3^a^
iPeak (mmol/L)^6^	3.68 ± 0.22^a^	2.72 ± 0.21^c^	3.14 ± 0.23^b^	3.60 ± 0.25^a^

^1^*All values are presented as mean* ± *SEM (n* = *22). Statistical analyses were performed using ANOVA followed by Tukey's post hoc test. Means within a row that do not share the same superscript letters (a, b, c) differ significantly (P*<*0.05)*.

^2^*Fasting glucose concentrations were measured prior to the start of the experimental day, immediately before intake of the first tablets (0 min)*.

^3^*Incremental changes from fasting in blood glucose concentrations at 90 min (i.e., immediately before the standardized breakfast)*.

^4^*Incremental blood glucose area under the curve (iAUC) calculated for the entire experimental period (0–240 min)*.

^5^*Incremental blood glucose area under the curve (iAUC) calculated for the postprandial period (90–240 min) following the standardized breakfast*.

^6^*Maximum incremental increase in blood glucose concentration during the test day, based on the individual peak rise from baseline*.

*iAUC, incremental area under the curve; iPeak, maximum increase at an individual test point during the test days*

Compared with placebo at all time points, ingestion of *P. ginseng* at 0 min (fasting) resulted in a reduction in postprandial blood glucose iAUC (90–240 min) by 29% (*P* < 0.001). In contrast, administration at 45 min or 90 min did not significantly affect postprandial iAUC compared with placebo (−12 and +2%, respectively; *P* > 0.05).

The iPeak glucose concentration after breakfast was significantly reduced when *P. ginseng* was provided at 0 and 45 min compared with placebo (−26%, *P* < 0.001 and −15%, *P* < 0.01, respectively). Furthermore, iPeak responses were lower when *P. ginseng* was administered at 0 or 45 min compared with administration at 90 min (−24%, *P* < 0.001 and −13%, *P* < 0.05, respectively). A more pronounced reduction was observed when ginseng was supplied at 0 min compared with 45 min (*P* < 0.05).

No differences in fasting blood glucose concentrations were detected between test days prior to the start of the experiment (*P* > 0.05) or immediately before breakfast (at 90 min; *P* > 0.05).

### Serum insulin

Significant main effects of *P. ginseng* treatment on serum insulin concentrations were observed during the postprandial period (90–240 min; *P* < 0.05), with no significant treatment^*^time interaction (*P* > 0.05; Figure 2B, Table 4).

**Table 4:** Fasting and incremental serum insulin responses under four experimental conditions: 1) placebo consumed at all preload time points (0, 45, and 90 min); 2) *Panax ginseng* administered at 0 min (with placebo at 45 and 90 min); 3) *Panax ginseng* administered at 45 min (with placebo at 0 and 90 min); 4) *Panax ginseng* administered at 90 min (with placebo at 0 and 45 min)^1^.

**Variable**	**Placebo at all time points**	**Ginseng at 0 min**	**Ginseng at 45 min**	**Ginseng at 90 min (at start of breakfast)**
Fasting insulin concentrations (nmol/L)^2^	0.035 ± 0.004^a^	0.041 ± 0.004^a^	0.039 ± 0.004^a^	0.036 ± 0.005^a^
At 90 min (nmol/L)^3^	−0.003 ± 0.003^a^	−0.013 ± 0.003^a^	−0.012 ± 0.003^a^	−0.011 ± 0.003^a^
iAUC 0–240 min (nmol^*^min/L)^4^	18.8 ± 2.9^a^	14.2 ± 2.5^b^	14.7 ± 2.1^b^	16.8 ± 2.7^ab^
iAUC 90–240 min (nmol^*^min/L)^5^	18.6 ± 2.9^a^	14.1 ± 2.5^b^	14.5 ± 2.1^b^	16.8 ± 2.7^ab^
iPeak (nmol/L)^6^	0.26 ± 0.03^a^	0.20 ± 0.02^c^	0.22 ± 0.02^bc^	0.24 ± 0.03^ab^

^1^*All values are presented as mean* ± *SEM (n* = *22). Statistical analyses were performed using ANOVA followed by Tukey's post hoc test. Means within a row that do not share the same superscript letters (a, b, c) differ significantly (P*<*0.05)*.

^2^*Fasting insulin concentrations were measured prior to the start of the experimental day, immediately before intake of the first tablets (0 min)*.

^3^*Incremental changes from fasting in blood insulin concentrations at 90 min (i.e., immediately before the standardized breakfast)*.

^4^*Incremental insulin area under the curve (iAUC) calculated for the entire experimental period (0–240 min)*.

^5^*Incremental insulin area under the curve (iAUC) calculated for the postprandial period (90–240 min) following the standardized breakfast*.

^6^*Maximum incremental increase in blood insulin concentration during the test day, based on the individual peak rise from baseline*.

*iAUC, incremental area under the curve; iPeak, maximum increase at an individual test point during the test days*.

Postprandial insulin iAUC following the standardized breakfast (at 90–240 min) was significantly reduced when *P. ginseng* was administered at 0 or 45 min compared with placebo (−24%, *P* < 0.001 and −23%, *P* < 0.01, respectively). Similarly, the maximum incremental peak insulin concentration (iPeak) was significantly lower after ginseng intake at 0 or 45 min compared with placebo (−23%, *P* < 0.001 and −15%, *P* < 0.01, respectively).

Furthermore, ginseng ingestion at 0 min resulted in a significantly lower iPeak compared with ginseng provided at 90 min (−17%, *P* < 0.01). No differences in fasting insulin concentrations were observed between test days (*P* > 0.05) or immediately prior to breakfast (90 min; *P* > 0.05).

In the Supplemental File 3, results regarding b-glucose and s-insulin responses are displayed based on absolute (actual) concentrations.

## Discussion

In the current study, preload time effects of *P. ginseng* on postprandial blood glucose and serum insulin concentrations were evaluated in healthy normal weight participants. Previous interventions have shown contradictory results in this regard [e.g. (17, 18)], which can be suggested partly be due to the choice of administration time of ginseng before the glucose challenge. Information from RCT in healthy participants investigating preload time effects of ginseng are scarce. In comparison to *P. quinquefolius* (American ginseng) and *P. notoginseng* (Chinese ginseng), effects of *P. ginseng* on glucose regulation in acute meal studies has been less investigated. Evaluation of time-dependent effects are thus crucial when judging possible health benefits of ginseng with respect to preventive potential against CMD, which reinforces the importance of this study.

The *P. ginseng* preload times points investigated in this study were 90 min, 45 min and directly before breakfast. The beneficial effect on glucose tolerance was demonstrated by reduced postprandial glucose- and insulin concentrations only after a sufficient preload time before the meal consumption, and was most pronounced when ginseng was provided 90 min prior to the meal. A preload of *P. Ginseng* taken 45 min prior to breakfast also elicited improved postprandial glucose tolerance, but in comparison to the 90 min preload the effects were less pronounced. The results from the current study do not support any effects of *P. ginseng* on glucose tolerance when ginseng is provided directly prior to the commencement of a meal. Previously, it was observed that *P. ginseng* (3 g) consumed 60 min prior to a standardized meal with white bread (50 g available starch) improved postprandial glucose regulation in healthy participants (17). On the contrary, no effects on postprandial blood glucose concentrations were observed in healthy individuals when *P. ginseng* (3 g) was administered 40 min prior to a glucose challenge (50 g) (19), or when healthy participants ingested *P. quinquefolius* (3 g) 20 min, 10 min or, in accordance to the results in the current study, immediately prior to the glucose load (50 g) (15).

The current study contributes novel findings by showing that, beyond reducing postprandial glucose responses, *Panax ginseng* also attenuates postprandial insulin responses. This pattern is consistent with prior reports suggesting that ginseng's glucoregulatory effects arise from improved insulin sensitivity rather than a secretagogue mechanism. Improved insulin sensitivity, as assessed by the insulin sensitivity index (ISI), was previously reported following a ginseng preload even when individual postprandial glucose and insulin concentrations did not decrease (19).

Furthermore, our work advances the field on two additional fronts. First, it is, to our knowledge, the first trial to evaluate a 90 min *P. ginseng* preload within a rigorously controlled meal study. Second-and most relevant for prevention—it focuses on *healthy, middle-aged, normal-weight* adults, a population in which attenuating postprandial excursions is particularly relevant. The observed reductions in both postprandial glucose and insulin therefore suggest that *P. ginseng* may support better postprandial regulation in a key prevention window, with potential long-term implications for cardiometabolic risk and type 2 diabetes.

Results from the current and previous studies thus indicate that the time between the preload of ginseng and the meal must be sufficiently long for achieving a significantly improved postprandial glucose tolerance. Consequently, 3, 6 or 9 g *P. quinquefolius* consumed as a preload 40, 80 or 120 min prior to a glucose challenge (25 g), improved postprandial glucose regulation in healthy participants (20). The preload time effects of ginseng may however differ between healthy and T2D. Thus, no differences in beneficial effects on postprandial glucose responses depending on preload time were detected when *P. quinquefolius* (3, 6, or 9 g) were administered to T2D patients at 120, 80, 40 or 0 min prior to a glucose challenger (25 g) (21), i.e. all preload time points resulted in improved postprandial glucose responses in the patients. The reason for the discrepancy with respect to time effects of ginseng between healthy and T2D are not described. One possible contributing mechanism could be an altered gastric motility and emptying rate in some diabetic patient, which possibly could interfere with time effects of substrates (22). Time-related effects of ginseng administration on glucose regulation can, however, be suggested to be an important determinant in healthy participants for health effects of *P. ginseng*.

In healthy participants, ground *P. quinquefolius* at doses of 1–3 g (15) or 3–9 g (20) resulted in reduced postprandial glucose responses, assessed by iAUC following a glucose challenge, regardless of dose size. In the presently described study, the test dose of *P. ginseng* was based on ginseng extract containing in total 32 mg ginsenosides, which equals 1.4 g ginseng root. This quantity corresponds to a common dose of ginseng supplementation consumed in the Western world (about 1–2 g per day).

Despite inconsistent results in acute studies on glucose regulation, both Asian Ginseng and American Ginseng, have been proposed to have several cardiometabolic health effects, e.g. improve fasting blood glucose concentration [reviewed in (9)], improve insulin resistance (animal-models) (23), and to have anti-inflammatory properties (animal-models) (11). The underlying mechanisms are not fully elucidated, and due to the wide range of potential bioactive ginsenosides, and other bioactive components that can be found in a minor extent in ginseng, the mechanisms whereby ginseng elicits metabolic health effects are probably multifactorial. Underlying mechanisms may also differ between acute effects and long-term effects of ginseng. However, one mechanism that has been proposed with respect to improved glucose tolerance, at least in longer-term studies, is related to increased plasma membrane translocation of GLUT4 and glucose uptake in skeletal muscle, and an upregulated expression of GLUT2 in the liver (24–26). An additional mechanism suggested to be involved in glucose regulatory effects of ginsenosides is an inhibitory effect on SGLT1, resulting in delayed glucose absorption in the intestine, which potentially contribute to attenuate postprandial glucose concentrations (26, 27).

Due to poor bioavailability in the upper gut, a significant proportion of ginsenosides enter colon and are potentially available for biotransformations by the gut bacteria. It has been suggested that compounds formed in colon may contribute to health effects on glucose regulation via inhibition of SGLT1 (27). It must be noted though, that the effects on glucose regulation in the current study were observed in an acute postprandial period after a meal consumed 90 min after intake of *P. Ginseng*, thus, the time elapsed between intake of ginseng and the observed effects is too short to claim involvement of colonic event as main underlying mechanisms.

This study has some limitations that should be considered. First, the extraction solvent used for the *Panax ginseng* preparation (aqueous vs. alcohol-based) was not disclosed by the manufacturer, which may restrict direct comparison with studies using explicitly defined extraction media. However, the preparation was standardized for total ginsenoside content, and the predominant ginsenosides were characterized. Second, health status, including absence of hypertension and food allergies, was based on self-reported information rather than clinical verification; nevertheless, baseline fasting glucose, insulin, and HOMA-IR values were within normal ranges, indicating a metabolically healthy cohort. Third, although the sample size was adequate for the prespecified primary outcomes, it was not powered for subgroup analyses by sex, and the unequal sex distribution (16 women, six men) further limits inference on potential sex-specific effects. Fourth, natural variation in ginsenoside profiles between ginseng plants may contribute to batch-to-batch variability and potentially to differences in physiological effects, which should be considered when comparing results across studies (28). Finally, the study was not designed to evaluate safety or toxicity; however, no adverse events were reported during the study visits.

Despite these limitations, the study has several strengths. The randomized, double-blind, placebo-controlled crossover design reduced inter-individual variability and increased internal validity. Experimental conditions were highly standardized with respect to diet, fasting, and physical activity, minimizing potential confounding of postprandial responses. Moreover, the within-subject comparison of multiple preload time points and the simultaneous assessment of glucose and insulin responses allowed a direct evaluation of timing-dependent effects and mechanistic interpretation in a prevention-relevant population.

In summary, *P. ginseng* improved postprandial glucose regulation when consumed 90 min before a meal, with smaller effects at 45 min and none immediately before eating. Concomitantly lower postprandial insulin responses support an insulin-sensitivity-driven acute effect rather than a secretagogue mechanism. Together, these findings suggest that ginseng preload may help attenuate postprandial glucose and insulin responses in a prevention-relevant population healthy population. While the study was acute in design, the observed improvements in postprandial regulation may have potential relevance for long-term cardiometabolic risk, including T2D. Further research is needed to confirm these effects and to determine optimal timing, dose-response relationships, and applicability across broader populations.

## Data Availability

The raw data supporting the conclusions of this article will be made available by the authors, without undue reservation.

## Supplementary material

Supplementary File 1 CONSORT 2010 Checklist for randomized crossover trials.

Supplementary File 2 CONSORT flow diagram.

Supplementary File 3 Supplementary Table S1 (glucose and insulin concentrations displayed with actual values), Supplementary Figure S1 (glucose curves with use of actual concentrations), Supplementary Figure S2 (insulin curves with use of actual concentrations).
