# Supplementary material for: Preload time-dependent effects of Panax ginseng on postprandial glucose tolerance. A randomized controlled study in healthy middle-aged participants
Source: Front Nutr. 2026 Feb 20;13:1759162. doi: 10.3389/fnut.2026.1759162 (PMC12962915; doi:10.3389/fnut.2026.1759162)
Supplement: Supplementary File 1 — CONSORT 2010 Checklist for randomized crossover trials. [file Data_Sheet_1.doc]

*Supplementary file 1*


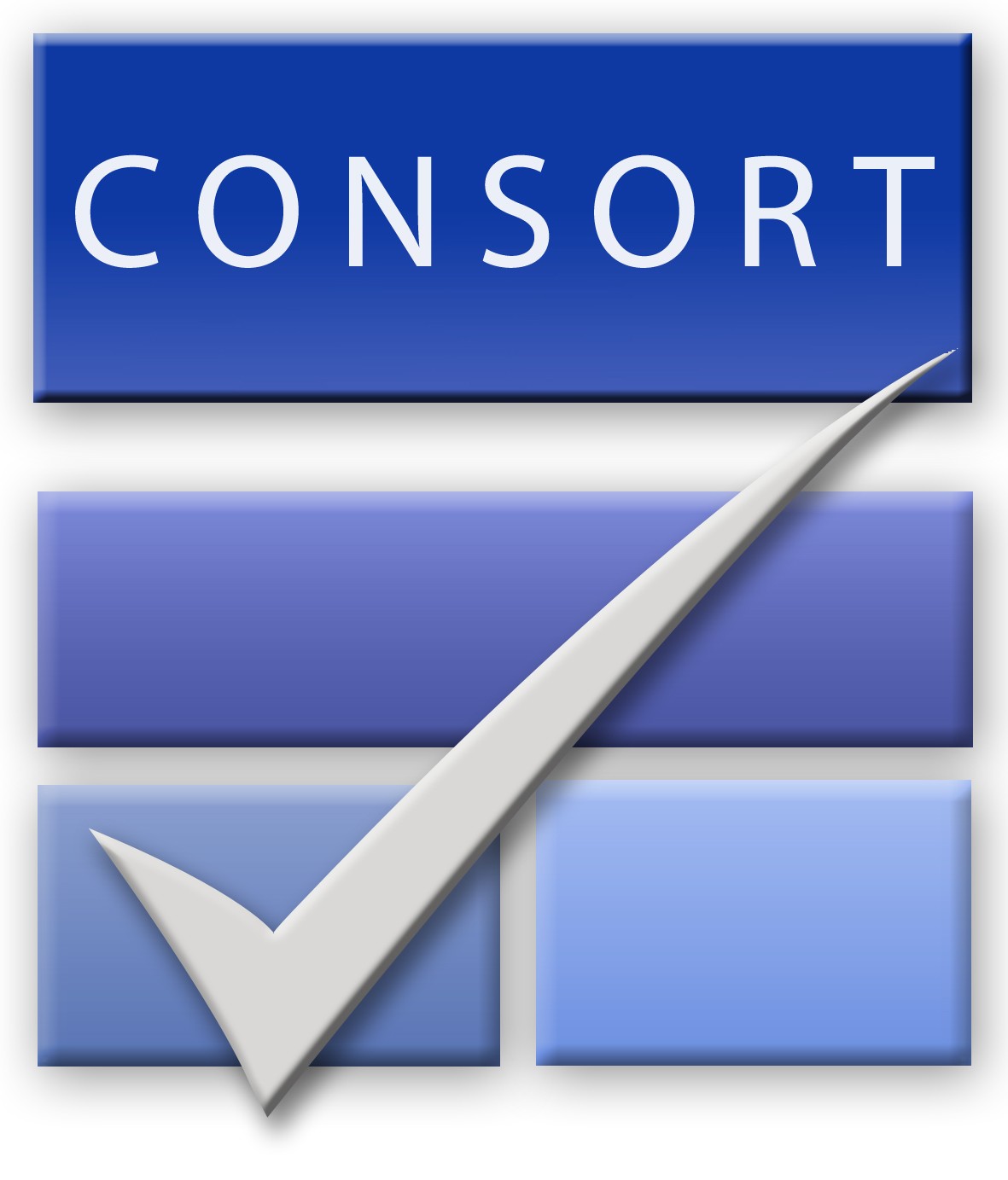
CONSORT 2010 checklist of information to include when reporting a randomised trial*

| Section/Topic | Item No | Checklist item | Reported on page No |
| --- | --- | --- | --- |
| Title and abstract | | | |
|  | 1a | Identification as a randomised trial in the title | 1 |
|  | 1b | Structured summary of trial design, methods, results, and conclusions (for specific guidance see CONSORT for abstracts) | 1 |
| Introduction | | | |
| Background and objectives | 2a | Scientific background and explanation of rationale | 2-4 |
|  | 2b | Specific objectives or hypotheses | 4 |
| Methods | | | |
| Trial design | 3a | Description of trial design (such as parallel, factorial) including allocation ratio | 4 (Study design and Procedure) |
|  | 3b | Important changes to methods after trial commencement (such as eligibility criteria), with reasons | Not applicable |
| Participants | 4a | Eligibility criteria for participants | 3 (Test participants) |
|  | 4b | Settings and locations where the data were collected | 4 (Study design and Procedure) |
| Interventions | 5 | The interventions for each group with sufficient details to allow replication, including how and when they were actually administered | 3-4 (the test and placebo products) |
| Outcomes | 6a | Completely defined pre-specified primary and secondary outcome measures, including how and when they were assessed | 5 (Calculation and statistical methods) |
|  | 6b | Any changes to trial outcomes after the trial commenced, with reasons | Not applicable |
| Sample size | 7a | How sample size was determined | 5 (Calculation and statistical methods) |
|  | 7b | When applicable, explanation of any interim analyses and stopping guidelines | Not applicable |
| Randomisation: |  |  | 5 (Calculation and statistical methods) |
| Sequence generation | 8a | Method used to generate the random allocation sequence |  |
|  | 8b | Type of randomisation; details of any restriction (such as blocking and block size) | 5 |
| Allocation concealment mechanism | 9 | Mechanism used to implement the random allocation sequence (such as sequentially numbered containers), describing any steps taken to conceal the sequence until interventions were assigned | 3-4 (identical placebo) |
| Implementation | 10 | Who generated the random allocation sequence, who enrolled participants, and who assigned participants to interventions | **Sequence:** blinded study investigator. **Enrollment:** blinded study investigator. **Assignment:** blinded lab assistant using pre‑packaged, code‑labeled, visually identical tablets. |
| Blinding | 11a | If done, who was blinded after assignment to interventions (for example, participants, care providers, those assessing outcomes) and how | See q 10 |
|  | 11b | If relevant, description of the similarity of interventions | 3-4 (Identival tablets) |
| Statistical methods | 12a | Statistical methods used to compare groups for primary and secondary outcomes | 5 (Calculation and statistical methods) |
|  | 12b | Methods for additional analyses, such as subgroup analyses and adjusted analyses | Not applicable |
| Results | | | |
| Participant flow (a diagram is strongly recommended) | 13a | For each group, the numbers of participants who were randomly assigned, received intended treatment, and were analysed for the primary outcome | 3, all 22 participants enrolled were analysed for the primary outcome |
|  | 13b | For each group, losses and exclusions after randomisation, together with reasons | None, see above |
| Recruitment | 14a | Dates defining the periods of recruitment and follow-up | 4 |
|  | 14b | Why the trial ended or was stopped | 4, completed as planned |
| Baseline data | 15 | A table showing baseline demographic and clinical characteristics for each group | Table 1 |
| Numbers analysed | 16 | For each group, number of participants (denominator) included in each analysis and whether the analysis was by original assigned groups | 22, crossover, all participants |
| Outcomes and estimation | 17a | For each primary and secondary outcome, results for each group, and the estimated effect size and its precision (such as 95% confidence interval) | 6, Tables 3–4; Figures 2A–B |
|  | 17b | For binary outcomes, presentation of both absolute and relative effect sizes is recommended | Not applicable |
| Ancillary analyses | 18 | Results of any other analyses performed, including subgroup analyses and adjusted analyses, distinguishing pre-specified from exploratory | Supplementary Figures S1–S2; Table S1 |
| Harms | 19 | All important harms or unintended effects in each group (for specific guidance see CONSORT for harms) | 9, no adverse events |
| Discussion | | | |
| Limitations | 20 | Trial limitations, addressing sources of potential bias, imprecision, and, if relevant, multiplicity of analyses | 8-9 |
| Generalisability | 21 | Generalisability (external validity, applicability) of the trial findings | 9 |
| Interpretation | 22 | Interpretation consistent with results, balancing benefits and harms, and considering other relevant evidence | 9 |
| Other information | | |  |
| Registration | 23 | Registration number and name of trial registry | ClinicalTrials.gov (Identifier: NCT02392819) |
| Protocol | 24 | Where the full trial protocol can be accessed, if available | The full trial protocol is available from the corresponding author upon reasonable request |
| Funding | 25 | Sources of funding and other support (such as supply of drugs), role of funders | 9, Antidiabetic Food Centre, a VINNOVA VINN Excellence Center at Lund University, funded the execution of the study |

*We strongly recommend reading this statement in conjunction with the CONSORT 2010 Explanation and Elaboration for important clarifications on all the items. If relevant, we also recommend reading CONSORT extensions for cluster randomised trials, non-inferiority and equivalence trials, non-pharmacological treatments, herbal interventions, and pragmatic trials. Additional extensions are forthcoming: for those and for up to date references relevant to this checklist, see [www.consort-statement.org](http://www.consort-statement.org/).
