# Supplementary material for: Preload time-dependent effects of Panax ginseng on postprandial glucose tolerance. A randomized controlled study in healthy middle-aged participants
Source: Front Nutr. 2026 Feb 20;13:1759162. doi: 10.3389/fnut.2026.1759162 (PMC12962915; doi:10.3389/fnut.2026.1759162)
Supplement: Supplementary File 2 — CONSORT flow diagram. [file Data_Sheet_2.doc]

Supplementary file 2

**Allocation**

**Analysis**

**Follow-Up**

**Enrollment**

Assessed for eligibility (n=22)

Excluded (n=0)

♦  Not meeting inclusion criteria (n=0)

♦  Declined to participate (n=0)

♦  Other reasons (n=0)

Analysed (N=22)
♦ Excluded from analysis (n=0)

Lost to follow-up (drop-out due to difficulties with compliance) (n=0)

Discontinued intervention (give reasons) (n=0)

Allocated to intervention (n=22)

♦ Received allocated intervention (n=22)

♦ Did not receive allocated intervention (give reasons) (n=0)

Participants (N=22) were randomly assigned, by use of Excel “RANDOM” formula,
to start with ginseng or placebo at time points 0 min (fasting), 45 min, 90 min (before start of the standardized breakfast). A cross-over design was applied. At each experimental day (4 days) the subjects received 2 tablets at each time point. At 3 of the test days the subjects received ginseng at one time point and placebo at the other two time points. At one test day they received placebo tablets at each time point.
