# Supplementary material for: Preload time-dependent effects of Panax ginseng on postprandial glucose tolerance. A randomized controlled study in healthy middle-aged participants
Source: Front Nutr. 2026 Feb 20;13:1759162. doi: 10.3389/fnut.2026.1759162 (PMC12962915; doi:10.3389/fnut.2026.1759162)
Supplement: Supplementary File 3 — Supplementary Table S1 (glucose and insulin concentrations displayed with actual values), Supplementary Figure S1 (glucose curves with use of actual concentrations), Supplementary Figure S2 (insulin curves with use of actual concentrations). [file Data_Sheet_3.docx]

Supplementary file 3

# Supplementary Figures and Tables

## Supplementary Figures

**Figure S1.** Absolute (actual) blood glucose concentrations 0 - 240 min when placebo was consumed at all preload timepoints (placebo at fasting (0 min), 45 and 90 min), when *Panax ginseng* were provided at 0 min (placebo at 45 and 90 min), at 45 min (placebo at 0 and 90 min), and at 90 min (placebo at 0 and 45 min). A standardized breakfast was consumed directly after the intake of the tablets at 90 min. Concentrations are presented as mean ± SEM, n=22.

**Figure S2.** Absolute (actual) serum insulin concentrations 0 - 240 min when placebo was consumed at all preload timepoints (placebo at fasting (0 min), 45 and 90 min), when *Panax ginseng* were provided at 0 min (placebo at 45 and 90 min), at 45 min (placebo at 0 and 90 min), and at 90 min (placebo at 0 and 45 min). A standardized breakfast was consumed directly after the intake of the tablets at 90 min. Concentrations are presented as mean ± SEM, n=22.

## Supplementary Table

**Table S1.** Fasting and blood glucose and insulin responses under four experimental conditions: 1) placebo consumed at all preload time points (0, 45, and 90 min); 2) *Panax ginseng* administered at 0 min (with placebo at 45 and 90 min); 3) *Panax ginseng* administered at 45 min (with placebo at 0 and 90 min); 4) *Panax ginseng* administered at 90 min (with placebo at 0 and 45 min) ^1^.

|  | **Placebo at all test points** | **Ginseng at 0 min** | **Ginseng at  45 min** | **Ginseng at 90 min** |  |
| --- | --- | --- | --- | --- | --- |
| ***Blood glucose:*** ***Results based on actual (total (t)) glucose concentrations*** | | | | | |
| Fasting glucose concentrations (mmol/L)^2^ | 5.3 ± 0.08^a^ | 5.5 ± 0.09^a^ | 5.3 ±0.09^a^ | 5.3 ±0.08^a^ |  |
| 90 min (mmol/L)^3^ | 5.4 ± 0.1^a^ | 5.3 ± 0.1^a^ | 5.3 ± 0.1 ^a^ | 5.2 ± 0.1^a^ |  |
| tAUC 0-240 min (mmol*min /L)^4^ | 1522 ± 32^a^ | 1470 ± 30^b^ | 1495 ± 29^ab^ | 1506 ± 34^ab^ |  |
| tAUC 90-240 min (mmol*min /L)^5^ | 1035 ±28^a^ | 977 ± 24^b^ | 1008 ± 24^ab^ | 1022 ± 29^a^ |  |
| tPeak (mmol/L)^6^ | 9.01 ± 0.23^a^ | 8.17 ± 0.21^b^ | 8.45 ± 0.23^b^ | 8.87 ± 0.25^a^ |  |
| ***Serum insulin:*** ***Results based on actual (total (t)) insulin concentrations*** | | | | | |
| Fasting (0 min) (nmol/L)^2^ | 0.035 ± 0.004^a^ | 0.041 ± 0.004^a^ | 0.039 ± 0.004^a^ | 0.036 ± 0.005^a^ |  |
| 90 min (directly prior to^3^ the breakfast) (nmol/L) | 0.032 ± 0.003^a^ | 0.027 ± 0.003^a^ | 0.027 ± 0.003^a^ | 0.025 ± 0.003^a^ |  |
| tAUC 0–240 (min*nmol/L)^4^ | 26.3 ± 3.3^a^ | 22.7 ± 2.9^b^ | 23.0 ± 2.6^b^ | 24.4 ± 3.3^ab^ |  |
| tAUC 90–240 (min*nmol/L)^5^ | 23.4 ± 3.1^a^ | 19.7 ± 2.7^b^ | 20.1 ± 2.4^b^ | 21.7 ± 3.0^ab^ |  |
| tPeak (nmol/L)^6^ | 0.29 ± 0.03^a^ | 0.23 ± 0.02^c^ | 0.25 ± 0.02^bc^ | 0.28 ± 0.03^ab^ |  |

^1^ All values are presented as mean ± SEM (n = 22). Means within a row that do not share the same superscript letters (a, b, c) differ significantly (*P* < 0.05; ANOVA followed by Tukey’s post hoc test). ^2^ Fasting glucose and insulin concentrations were measured prior to the start of the experimental day, immediately before intake of the first tablets (0 min). ^3^ Blood glucose and serum insulin concentrations at 90 min (i.e., immediately before the standardized breakfast). ^4^ Area under the curves based on actual concentrations (tAUC) calculated for the entire experimental period (0–240 min). ^5^ Areas under the curve (tAUC) calculated for the postprandial period (90–240 min) following the standardized breakfast. ^6^ Maximum blood glucose and serum insulin concentration at the test day, based on the individual peak concentrations. tAUC, total (actual) area under the curve; tPeak, maximum concentration at an individual test point during the test days.
